# African-American Women’s Perceptions and Experiences About Breastfeeding

**DOI:** 10.3389/fpubh.2015.00273

**Published:** 2015-12-21

**Authors:** Cecilia S. Obeng, Roberta E. Emetu, Terry J. Curtis

**Affiliations:** ^1^School of Public Health, Indiana University, Bloomington, IN, USA; ^2^Department of Health Sciences, College of Health and Human Development, California State University Northridge, Northridge, CA, USA; ^3^Indiana Black Breastfeeding Coalition, (http://indianablackbreastfeedingcoalition.com)

**Keywords:** breast feeding, African-American women, perceptions, health benefits, newborns, community-based support, educational status

## Abstract

There are health benefits to breastfeeding for both mothers and their children. The preventive health effects of breastfeeding continue into adulthood, lowering rate of various chronic illnesses. African-American women, especially of lower socioeconomic status, are less likely to breastfeed in comparison to their racial and ethnic counterparts. The purpose of this study is to explore how African-American women experience breastfeeding in the early stages of postpartum care. Two focus groups (*N* = 20, 10 in each group) were conducted with African-American mothers. Results revealed that participants felt that there were health benefits to breastfeeding, and organizations such as Women, Infants, and Children (WIC) provided support. However, participants stated that lack of information, negative perceptions, and unforeseen circumstances were barriers to breastfeeding. This study proposes support and interventions for this group to increase breastfeeding among this population.

## Introduction

The World Health Organization (1) recognizes breastfeeding as the best source of nourishment for newborns and infants. It is recommended that new mothers exclusively breastfeed their newborn for a minimum of 6 months and incorporate complementary foods in conjunction with breast milk after that point (1–3). Breastfeeding is an essential part of postnatal childcare and is associated with optimal benefits for both the mother and her child. One of the goals of *Healthy People 2020* is to increase the percentage of women who breastfeed up to 60% (4). In the United States (U.S.), African-American women have the lowest rate of breastfeeding in comparison to their White, Asian, Latino, and other racial/ethic counterparts (5). The racial/ethic disparity surrounding breastfeeding is even lower among African-Americans who reside in low-income areas (6, 7).

### Health Benefits of Breastfeeding

As noted in the literature, the advantages of breastfeeding include nutritional, immunological, cognitive, and medical benefits for newborns and infants (8), including fewer hospital visits and better health and developmental outcomes (9). Breast milk provides protein, fat, carbohydrates, liquid, and other nutrients (10). Apart from the nutritional value provided by breast milk, breastfeeding is associated with lower rates of obesity, cardiovascular diseases, and certain cancers (11–13). Breastfeeding also improves the immune system of infants because it contains antibodies that can assist in combating harmful bacteria and viruses (14). In addition, breastfeeding reduces the risk for ear infections, allergies, and respiratory conditions, such as asthma (15–17). Various studies have noted the link between breastfeeding and a reduction in sudden infant death syndrome (SIDS) (18, 19). The benefits of breastfeeding are vast for acute and chronic conditions for one’s offspring.

Apart from children, new mothers can gain immensely from breastfeeding. First, there are several postpartum benefits; for instance, studies have shown that hormones stimulated during breastfeeding can assist with returning the uterus back to its original size (20). By breastfeeding, the mother has a boost in her metabolism and is able to accelerate the loss of maternal weight (21). Second, breastfeeding can protect against chronic infections, such as ovarian and breast cancer (22). Furthermore, Sturtz et al. (23) found that African-American women were more likely to develop breast cancer in comparison to White women, and electing not to breastfeed was a determinant for breast cancer development.

Based on epidemiological data, African-Americans have the highest rates of cardiovascular disease, obesity, diabetes, and hypertension (24), and breastfeeding has been proven to combat, prevent, and reduce the rates of some of these chronic conditions (9, 11, 12, 21, 25). Breastfeeding is a preventive health behavior that is cultural, and among low-income African-American women, it is poorly understood. Therefore, further research is needed to understand and explore how African-American women experience breastfeeding.

### Determinants Contributing to Decisions to Not Breastfeed

Low breastfeeding rates can negatively impact the health of mothers and their babies. The literature on breastfeeding has reported barriers and constraints that contribute to the low rates of breastfeeding among African-American women. Hannon et al. (26) found that African-American women decisions to breastfeed were swayed by their perceptions of the benefits of breastfeeding, their perceptions of the problems with breastfeeding, and influential people in their lives. Historically, African-American women were utilized as “wet nurses,” where women were required to breastfeed children of households where they rendered service as caregivers (27). Perhaps, after the “wet nurse” era ended women within the African-American community perceived breastfeeding as a symbol of powerlessness or objectification. Lewallen and Street (28) conducted a study where they found that African-American women choose not to breastfeed due to lack of support from their mothers, grandmothers, and significant others; often times, these relatives did not breastfeed during their postpartum years.

According to the literature, lack of resources is another determinant that could contribute to the low rates of breastfeeding among African-American women (29–32). Miscommunication, lack of information, and inadequate training could deter a new mother from breastfeeding (29, 30). Some women experience pain during breastfeeding, at times it is difficult for the baby to latch correctly, and women may perceive that they have insufficient milk supply (31–33). Chantry et al. (33) found that in-hospital formula use increased early breastfeeding cessation. Therefore, without adequate training and with a convenient supply of synthetic milk in hospitals, women might develop trust in formula and become more comfortable not breastfeeding (34).

Environmental factors also contribute to African-American women’s decision to breastfeed. Li et al. (35) noted that low-income African-American women need extra support in order to breastfeed publicly and in their places of employment. There is a negative perception with breastfeeding and the continuation of employment, leading to premature breastfeeding cessation (36). Chuang et al. (37) reported that women were more likely to breastfeed if they had maternal leave; however, upon returning to work most ceased to breastfeed. One of the goals of *Healthy People 2020* is to increase the proportion of employers by 38% that have worksite lactation support programs (38). Employer support is an important factor for breastfeeding initiation and continuation, especially for low-income women who may work part-time or do not have the benefit of maternity leave. Therefore, it should be a public health goal to explore ways to increase the initiation and duration of breastfeeding among African-American women through the context of their experiences, desires, and needs.

### Purpose of Study

The low rates of breastfeeding among African-American women are poorly understood. Therefore, the purpose of this study is to explore and further understand how African-American women experience breastfeeding as well as which constructs could initiate and promote breastfeeding in the early stages of postpartum care.

## Materials and Methods

This study was conducted after approval from an Institutional Review Board. Participation in this research was voluntarily.

African-American mothers between the ages of 20 and 40 were recruited by the Indiana Black Breastfeeding Coalition (IBBC) to participate in a focus group about their perceptions of breastfeeding. The women were either first time mothers or mothers with children. Participants were split into two groups of 10 women. A research team facilitated the focus groups. Participants received a $40 Target gift card at the end of the session. The focus groups were conducted at a community center in the Midwest. Participants were of lower and middleclass status and were recruited primarily through flyer advertisements at local urban settings.

The purpose of the focus group sessions was to further understand perceptions, opinions, beliefs, attitudes, and the family dynamics on breastfeeding among African-American women. Upon arrival, participants were asked to sign-in and complete a questionnaire about demographic characteristics, such as age, highest education level, employment status, and relationship status. After the collection of the demographic data, a preliminary open-ended questionnaire was administrated to assess the breastfeeding education level of participants. A facilitator from IBBC led a discussion about the history of breastfeeding and provided the correct answers to the preliminary questionnaire.

Throughout the focus group sessions, which typically lasted about 2 hours, women were encouraged to describe as a group both their physical (if they breastfed after previous childbirth) and psychosocial experiences, including their perception of breastfeeding. No attempt was made to limit their responses. The narratives were audio recorded, transcribed, and later analyzed into themes.

Analysis for this study began by reading through the data multiple times for familiarization of the data. Similar responses were grouped through comparison of participants’ answers. Themes were developed and name codes were assigned to individual themes. Verbatim responses were highlighted based on the categories identified to best illustrate the theme.

## Results

The results of the pre-assessment demographic information indicated that majority (*n* = 13) of the mothers were single. Nine had full time jobs, and the rest either worked part-time or were unemployed. Majority (*n* = 13) had completed an associate degree or had a lower level of education (see Table 1 for demographic characteristics).

**Table 1:** **Participants demographic**.

Participants	Age	Last grade in school completed	Are you employed?	Relationship status
1	30	11th Grade	No	Single, but in a relationship
2	31	2 years Technical degree or some college	Yes, part-time	Single, not married
3	34	2 years Technical degree or some college	Yes, full-time	Separated/divorced
4	34	Some graduate school or a graduate degree	Yes, full-time	Married
5	40	11th Grade	No	Separated/divorced
6	38	2 years Technical degree or some college	Yes, full-time	Single, not married
7	35	Some graduate school or a graduate degree	Yes, full-time	Married
8	36	4 years Bachelors’ degree	Yes, full-time	Married
9	25	4 years Bachelors’ degree	Yes, full-time	Single, but in a relationship
10	34	Some graduate school or a graduate degree	No	Married
11	33	2 years Technical degree or some college	Yes, part-time	Single, not married
12	33	2 years Technical degree or some college	No	Married
13	24	4 years Bachelors’ degree	Yes, part-time	Single, but in a relationship
14	20	2 years Technical degree or some college	No	Single, but in a relationship
15	26	High school diploma or GED	Yes, full-time	Single, not married
16	20	High school diploma or GED	No	Single, not married
17	27	Some graduate school or a graduate degree	Yes, full-time	Married
18	25	2 years Technical degree or some college	Yes, full-time	Married
19	32	2 year Technical degree or some college	Yes, full-time	Single, but in a relationship
20	29	2 years technical degree or some college	No	Single, not married

### Theme 1: Health Benefits

Some of the participants had positive intentions to breastfeed their children because they strongly believed that breastfeeding encouraged bonding for both mother and child. Participants suggested that the immunological health benefits of nursing and the long-term cognitive benefits were factors that should encourage mothers to nurse. They also stated that children who were breastfed were much brighter and attached emotionally than those who were not. Participants also identified human milk as natural, with the nutrients a child needs, and is the best for both the mother and her offspring. Participants also noted that mothers who breastfeed lost weight quicker in comparison to women that did not. Below are the excerpts that support the information stated above:

Healthy, the best thing for everybody, you and the baby.

You lose the weight way quicker … way quicker.

For me a huge reason is the immunological health benefits of nursing and the long-term cognitive benefits. Those or children that are nursed are much brighter and more attached emotionally than those that are not.

Human milk! It’s natural; it has all the components your body needs.

I read a study, if you breastfed you are less likely to get breast cancer later on.

### Theme 2: Lack of Information

Participants indicated that breastfeeding was not mentioned during their prenatal or postnatal medical visits. Thus, some participants did not receive any pertinent information about breastfeeding from any health professional. The lack of discussion about breastfeeding between healthcare providers and mothers contributed to decisions to not nurse their newborns. Participants suggested that including breastfeeding information in prenatal education would have been beneficial in teaching and providing education about the differences between human milk and milk substitutes. Supportive communication about breastfeeding would have assisted tremendously toward helping new mothers to breastfeed. Below are the excerpts that support the information stated above:

My OB/GYN didn’t talk about it. Outside of this, none.

They drew me to any doctor. And she [the physician] doesn’t really talk about it, which is weird because she’s Indian, Arabian. I would assume she would [discuss breastfeeding] because they do that in her culture, but she didn’t mention it.

I didn’t know about breastfeeding until I got pregnant. I had never heard about … they should let you know when you’re 13. Through health education classes. They should mention it. Why am I 24 and I’m just now just finding out what breastfeeding is, that’s kind of crazy.

### Theme 3: Negative Perceptions of Breastfeeding by Others

Many participants noted that lack of breastfeeding support and negativity in regards to breastfeeding were pronounced within their interpersonal relationships and public associations, which affected breastfeeding initiation or cessation. Participants indicated that their mothers, grandmothers, and women who attended their churches firmly stated that breastfeeding led to the sagging skin of the breast. Participants noted that breastfeeding initiation was difficult due to a lack of family role models that breastfeed. Others reported that they received free synthetic milk through Women, Infants, and Children (WIC), and these governmental agencies assisted in their decision to use formula. Participants who were employed emphasized that their supervisors and co-workers were either unsupportive or against breastfeeding. In addition, participants expressed shame from various people about breastfeeding publicly. Below are the excerpts that support the information stated above:

#### Lack of Family Support

This is my fourth pregnancy. I tried to breastfeed with my first but it was a terrible experienced so I stopped. But I think that was basically because I didn’t have any support doing it.

I made the choice in the beginning, because nobody in my family has done a whole lot of breastfeeding. They’re all pretty much against it.

My grandmother telling me that my breast would sag.

#### Lack of Support from Friends

I asked one of my friends why she didn’t breastfeed and she’s like “It’s incest.” I was like but the baby came through all of the other nasty gross parts, so … I didn’t even have an answer for that.

What I run into when I tell … friends that I, you know, I want to breastfeed, that I tried it before and it didn’t work out like I wanted it to but I still want to breastfeed with this child and I, uh, here a lot of people don’t see it as … you know, a lot of people see it as, uh, a lot of black people I be around sometimes see it kind of as perverted, and why you want to and they’ll say I don’t want nobody sucking on me, you know, and I’m like that’s, you know, but that’s how we’ve been taught. It’s not a normal thing or something that’s natural, you know.

#### Lack of Employer/Co-Worker Support

All of mine [Coworkers] were like you’re gonna go do that. You know breasts are suppose to be for sex.

They [employer] wanted me to pump in the bathroom, and I’m like I’m not doing that, you know. And so then they wanted me to pump in the closet.

My coworker was so anti-breastfeeding, she was not supportive. When I told her that I want to go pump and she was my boss, she made it really hard for me.

#### Lack Support Publicly

As far as doing it in public. I know one time I was at Wal-Mart and I was with my son, and I had one of those newbie raps, so you really couldn’t really tell I was breastfeeding and I’m walking through Walmart. But like you know this White lady walked up to me like “are you breastfeeding at Walmart” and I’m like yes, I am. Do you have a problem with that, I don’t even know you. She was like, “shouldn’t you go in the bathroom or something,” and I’m like okay Imma walk away from you cause you gonna make me loss my intelligence real quick.

I’m 31, and mostly everybody I know … it’s eleven of us … I think it’s only three of us that are gonna breastfeed. Everybody else is like well I don’t think it’s beneficial. I don’t wanna be out in a restaurant and have to do it. You know I’m telling them about the canapés and all that now.

### Theme 4: Organizational Support

Participants specified that the IBBC, WIC, St. Vincent’s Hospital, churches, and hospital lactation consultants helped them with breastfeeding education and support. Some participants noted that WIC gave resourceful breastfeeding information. Lactation consultants provided home visits to assure that children were positioned properly to the breast, and that mothers were breastfeeding correctly. Other community support groups provided classes that were beneficial for mothers. Below are the excerpts that support the information stated above:

It was really nice the support I got from my church.

I actually went to a birthing class at St. Vincent’s and they offered a lot. That class was an advocate for breastfeeding and a lot of things that are more natural. But honestly, if I hadn’t gone to the breastfeeding class with the black breastfeeding coalition I wouldn’t have gotten half of the information that I had.

And the lactation specialist from the hospital came too. I can’t remember if they came to my house or if I actually called them, either way I remembered getting support after I came home.

WIC gives out the pump, as long as you pump and only use four cans. They’ll only give you four cans of formula and the pump. But they won’t give you more than four cans.

### Theme 5: Unforeseen Circumstances of Breastfeeding

Research participants in this group stated that they experienced unforeseen problems when they attempted to breastfeed their children. One problem that participants faced was that some children were lactose intolerant; therefore, diary had to be extracted from the diet. Some noted that they were encouraged to take supplements to produce sufficient milk for their babies. Participants indicated that they were highly stressed from the lack of assistance in regards to the issues mentioned above. The excerpts below confirm participants concerns:

With my daughter I guess they said my production wasn’t enough. Um … so they had told me to start taking these supplements, but it still seems like my high production wasn’t coming up so I had to substitute for formula. I hated that, but you know I kept giving her the breast milk too.

And because she was lactose intolerant I had to take all dairy, that was everything that was dairy out of my diet. It was a question mark if I was able or willing to make that sacrifice for myself, but when I realized that again it was because of her and the attachment that she had to it, and I did. And you lose weight that much faster so it all worked out. She grew out of it, like very soon thereafter.

They were just like “Hurry up” and you know what? I’m still trying to learn this and they’re just like “Come on” and I realize that … but can you please calm your nerves because I’m a little stressed right now and they’re still a little tender. I’m tender here. Everything hurts. Can you please just show some compassion for my situation.

My problem was like I didn’t know- it was my first baby. I was “okay, breastfeeding is what I want to do”. And she comes in and basically she was like “This is your boob, this is your baby, mouth to boob”. And I was like “okay. What part of the boob goes into the baby’s mouth?” Like, I didn’t really know. And then it’s just like okay she’s on but is she done? How do I know she’s getting it? Is it coming out? I didn’t know what to ask. And then she’s like “When she stops put her on the other one”. And I was like okay. And she was sleep and I was still having her on there and my mom walks in like “What are you doing?” I’m like “Breastfeeding”. And she was like “The baby’s sleep”. And I was okay she’s sleeping and she’s done now so I called the lady back and she was like “You didn’t know how to – ” and I was like “No, I did not know how to take her off right.”

So when my sister went into labor and she had her baby and what not and the nurse was trying to show her how to breastfeed and stuff like that, it was just like you said. She was like “Do this or do that.”

And if you’re always in pain something’s not right.

I hear a lot of people saying it’s going to hurt and stuff.

## Discussion

Based on the above results, it is a viable assumption that mothers will breastfeed if they receive knowledge and information on the health benefits of breastfeeding from healthcare providers and professionals, lactation consultants, and hospital-based or community-based lactation clinicians. Also, it is self-evident that mothers need physical and emotional support from families, friends, and local community groups in order to initiate and continue to breastfeed through the recommended duration. Participants indicated that a lack of breastfeeding support was stressful. Thus, it is important for all stakeholders to use available tools (i.e., hospital-grade pumps) or strategies to encourage breastfeeding during and after hospitalization in order to ease the physical and emotional burden on mothers.

Support at the workplace was also a major factor that could have negatively affected breastfeeding. Participants who worked in places that were not supportive or hostile to breastfeeding did not feel comfortable breastfeeding their newborns or infants. Therefore, a change in attitude toward breastfeeding mothers, both at the workplace and in the home might garner support and consequently boost breastfeeding.

Instead of close relatives and friends instilling fear in mothers about how painful it is to breastfeed (as was the experience of some of the research participants), mothers should be encouraged to understand how they can receive professional advice about pain reduction, pain management, and understanding the differences between breast milk and synthetic milk. With support, the increased awareness of the health benefits of breastfeeding could assist mothers further in choosing to breastfeed. Emphasizing the idea of the gains of breastfeeding, such as bonding, immunological health benefits, and the long-term cognitive benefits to mothers may encourage them to decide in favor of breastfeeding no matter the problems they may encounter associated with breastfeeding.

Generally, without education, community-based support, and support from one’s social support system, mothers of newborns may either not initiate breastfeeding, decide not to exclusively breastfeed for the first six crucial months in a newborn’s life, or stop breastfeeding after a few days, weeks, or months. Not only will each of the above actions by mothers be detrimental to breastfeeding; it could potentially lead to newborns being denied essential food nutrients; a situation that could easily lead to morbidity and possible mortality as noted in the literature (9–12, 25).

For mothers, the adverse health effects of not breastfeeding are highly amplified in scholarly breastfeeding literature, linking breastfeeding non-adherence or cessation to weight gain and various chronic illnesses (20, 22, 23). It is concomitant to encourage mothers to initiate breastfeeding at the appropriate time, and continue through the duration of the recommended time with strong professional advice if needed. Also, encouragement is needed from one’s social support system since breastfeeding is beneficial to all stakeholders involved in raising the child.

Based on the participants’ comments and suggestions the following recommendations are made to increase breastfeeding and promote breastfeeding practice. Perhaps, an educational course or workshop would be valuable for families and significant others, which would disseminate knowledge about the benefits of breastfeeding, the differences between breast milk and formula, and the level of support a new mother needs to initiate breastfeeding and progress successfully. Leaders must also provide money to breastfeeding educators to develop clear messaging that will inform mothers about the health importance and emotional benefits of breastfeeding to both mothers and social support system. This is especially relevant for African-American mothers since breastfeeding rates are low among them.

In conclusion, this study has demonstrated the perception and experiences of breastfeeding among African-American women, in an urban setting, a population that are less likely to breastfeed. Interventions could be reached by addressing the concerns raised by the participants in this study.

## Author Contributions

CO and RE conducted the study and wrote the manuscript.

## Conflict of Interest Statement

The authors declare that the research was conducted in the absence of any commercial or financial relationships that could be construed as a potential conflict of interest.
